# Leopard sterilisation in India: The ecological and ethical costs of anthropocentric wildlife management

**DOI:** 10.1007/s13280-026-02473-0

**Published:** 2026-08-14

**Authors:** Pranay Arora

**Affiliations:** School of Geography and the Environment, University of Oxford, Oxford, UK

**Keywords:** Anthropocentric, Human–leopard conflict, Immuno-contraceptives

## Abstract

India’s rising human–leopard conflict has prompted the government to adopt immune contraceptives to manage leopard populations. The decision has sparked debate among wildlife experts and conservation organisations, government officials, and local communities. Here, I discuss the ecological and ethical implications of this intervention and highlight alternative, coexistence-based approaches that recognise both humans and wildlife as stakeholders within shared socio-ecological systems.

## Introduction

Human–wildlife conflict (HWC) has been recognised as a significant risk to conservation efforts and wildlife–community management globally. The challenge is particularly pronounced in developing economies, where rural households survive on narrow economic margins and the loss of livestock, crops, or human life can significantly affect community livelihoods, resulting in severe financial stress, debt accumulation, and food insecurity, thus heightening tensions (Braczkowski et al. 2023). India represents a key example of this dynamic, where a dense human population, expansion of urban and agro-environments, and success of decades-long conservation efforts have caused frequent interactions between humans and wildlife. Although coexistence with wildlife has deep historical and cultural roots in India, tolerance in modern-day contexts is increasingly shaped by livelihood dependence and economic costs of conflict. Consequently, human–wildlife conflict is becoming increasingly common in India, with the country projected to become a global hotspot of HWCs by 2070 (Ma et al. 2024). Of all the species involved in HWCs in India, Asian elephant (*Elephas maximus*), leopard (*Panthera pardus*), and tiger (*Panthera tigris*) constitute the most reported incidents (Anand and Radhakrishna 2017). Among these three, leopards present the most complex conflict profile due to their wide distribution, rising population, ecological adaptability, and frequent presence in human-dominated landscapes.

Between 2018 and 2022, leopard population in India grew at a rate of 1.08% per year. However, the growth rate was not equal everywhere with states like Maharashtra and Tamil Nadu experiencing a ~ 1.5% increase while the Shivalik Hills and Gangetic plains witnessed a 3.4% decline each year (Qureshi et al. 2024). While the rise in overall leopard population reflects success of conservation programmes, it was tainted by increasing human–leopard conflicts. These conflicts often involve livestock depredation and attacks on people, leading to resentment among the local population whose livelihoods are directly affected. Historically, the Government of India has managed human–leopard conflict through relocation, compensation, and, in extreme cases, lethal control; however, escalating conflict levels have prompted growing interest in science-based interventions. Consequently, in November 2025, the use of immuno-contraceptives was approved to manage leopard population in certain regions of India.

The approval to use immuno-contraceptives came after increased public outrage in villages around Junnar forest in the Pune district of Maharashtra following the death of 8 people in 2024 and 5 in 2025 due to leopard attacks. The Junnar region has historically experienced significant human–leopard conflict due to high leopard density and the expansion of sugarcane cultivation. Sugarcane has expanded across the region since it is a highly profitable cash crop in Maharashtra, supported by government-backed sugar co-operatives that provide assured procurement and prices, subsidised agricultural infrastructure, and direct employment (Lalvani 2008). These economic incentives have driven land-use transformation, bringing humans and leopards into frequent contact historically. The recent rise in conflicts was blamed on the crossing of forest’s leopard carrying capacity, forcing more leopards into sugarcane fields. The approved pilot plan involves administration of immuno-contraceptives on 125 female leopards in the region, using darts to trigger immune system responses that prevent reproduction without the need for surgical methods (Kulkarni 2025). Such an intervention carries significant implications which must be discussed within the socio-economic realities of the region before an on-ground implementation.

## Ecological and ethical implications

The use of immuno-contraceptives to manage wildlife populations is not novel and has been applied in species such as wild horses, African elephants, and urban deer (Kirkpatrick et al. 2011). However, the Indian government’s proposed pilot represents the first application of immuno-contraception on an apex predator, the leopard. Two main classes of immuno-contraceptives are used in wildlife management: porcine zona pellucida (pZP) vaccines, which inhibit fertilisation by preventing sperm–egg binding, and gonadotropin-releasing hormone (GnRH) vaccines, which suppress reproductive hormone production. Although these methods are often framed as non-lethal and reversible, their ecological consequences, particularly in large carnivores, remain poorly understood.

**Fig. 1:**
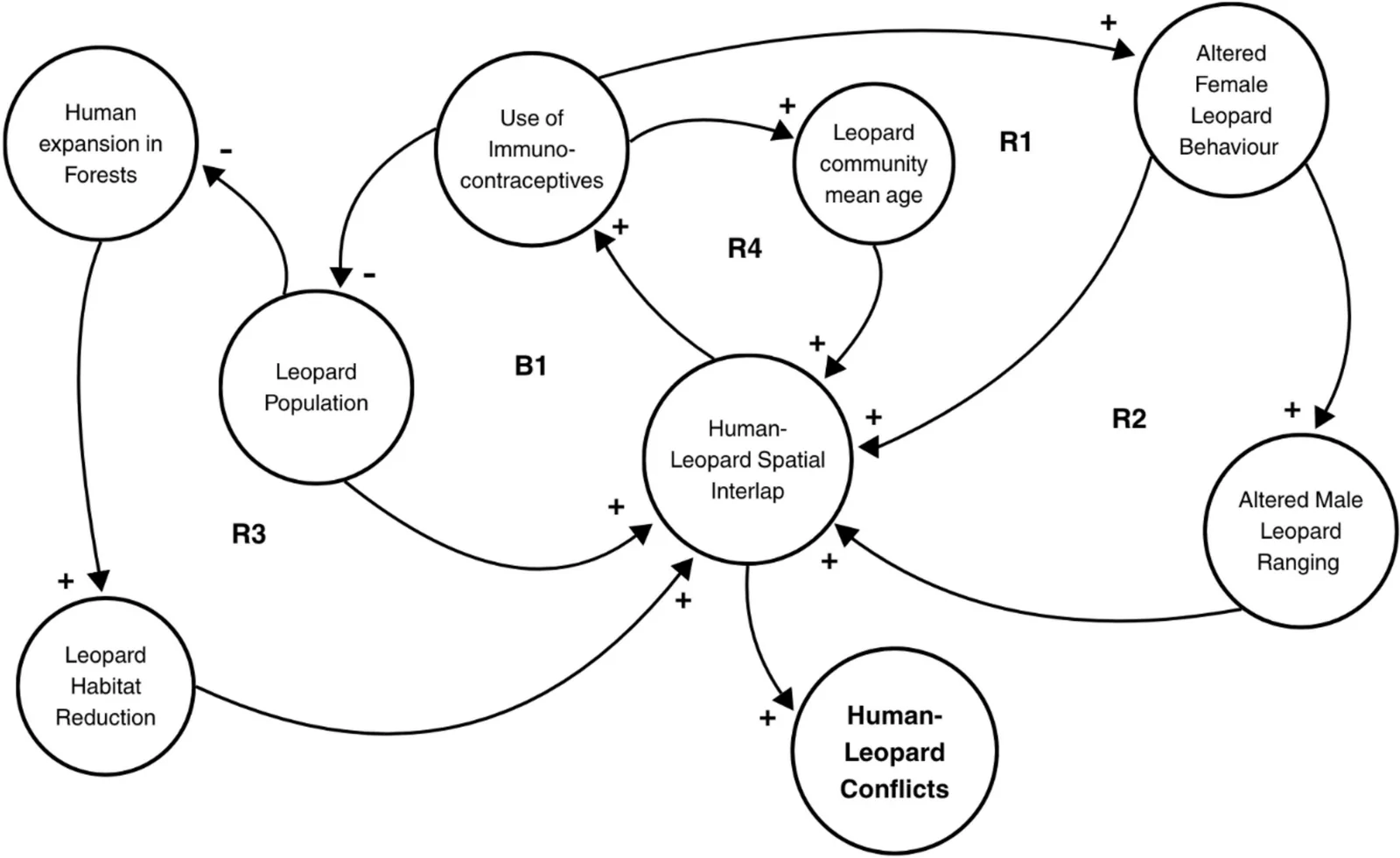
Causal loop diagram illustrating the potential direct and indirect effects of immuno-contraceptive use on leopard population management and human–leopard interactions. Positive links ( +) indicate reinforcing relationships, where an increase in one variable leads to an increase in the other, while negative links (−) indicate balancing relationships, where an increase in one variable leads to a decrease in the other. Reinforcing (R) and balancing (B) feedback loops highlight the potential dynamics of the system

Existing studies examining the effect of immuno-contraceptives on feral horse behaviour report inconsistent results, with some indicating no behavioural change following pZP treatment (Ransom et al. 2010), while others document increased female movement and altered mating behaviour (Nuñez et al. 2009). Similar inconsistencies have been reported across taxa, and no clear consensus exists regarding the influence of immuno-contraceptives on social dynamics in free-ranging wildlife. Such uncertainties are particularly concerning for leopards, whose spatial ecology and territorial behaviour are closely linked to reproduction. As a result, immuno-contraceptives have the potential to influence leopard populations through both demographic and behavioural pathways. Demographically, reduced reproduction could increase the proportion of older individuals who are more likely to venture into human habitats due to territorial pressures (Fig. 1). While behaviourally, if female leopards respond similarly to the increased movement observed in pZP-treated female feral horses, it could influence male territoriality, potentially leading to expanded ranges and more human–leopard encounters (Fig. 1). Thus, applying immuno-contraceptives on leopards at such a large scale requires prior research to understand their long-term effects on leopard population dynamics.

Use of immuno-contraceptives also does not address the primary drivers of conflict in the Junnar landscape: forest fragmentation resulting from expanding agriculture and urbanisation, along with establishment of alternative habitats in sugarcane fields. Sugarcane fields provide dense cover, abundant prey such as stray dogs, feral pigs and livestock, as well as irrigation canals that provide water all year round, creating suitable long-term habitat for leopards (Badhe and Jaybhaye 2024). Thus, even if localised fertility control successfully reduced leopard population in the fields, its effectiveness may get undermined by continued immigration of leopards from surrounding habitats, limiting it long-term effectiveness.

The programme also raises complex ethical questions. Although immuno-contraception is often presented as a humane alternative to lethal control, this framing overlooks potential welfare concerns including the stress from darting, possible side effects of hormonal disruption, and impacts on natural behaviour. From a governance perspective, such interventions risk normalising an approach which legitimises continued human expansion into wildlife habitats, rather than addressing the underlying drivers such as habitat loss, agricultural encroachment, and unregulated development. This could put the burden of coexistence disproportionately on wildlife as well as risk creating a reinforcing cycle where increasing human land-use intensifies conflicts, prompting greater reliance on population control measures that, in turn, legitimise further encroachment (Fig. 1). It also sets a broader precedent for conservation in India, where population control may increasingly be used to accommodate human land-use priorities, even in ecologically sensitive areas, as seen in development pressures around Sanjay Gandhi National Park, Mumbai (Lohia 2025).

Immuno-contraception also reflects an anthropocentric framework of conservation in which wildlife are primarily treated as populations to be regulated for human safety and convenience. While conservation is inherently anthropogenic, immuno-contraception differs fundamentally from other conservation measures such as establishing protected areas since it disrupts a species’ natural biological function, thus ignoring them as stakeholders in shared socio-ecological systems. Furthermore, in the case of leopards, whose presence in agricultural landscapes is largely a consequence of human-driven habitat transformation, controlling reproduction may obscure questions of responsibility and justice in conservation decision-making. Consequently, while immuno-contraceptive programmes may appear ethically preferable to lethal control, they raise concerns about how coexistence is defined and whose interests are prioritised in increasingly human-dominated landscapes.

At the same time, there are equally important ethical considerations for human communities. While the protection of leopards is a critical global conservation goal, it is important to ask whether that should come at the cost of local communities. In Junnar, dependence on sugarcane farming, an economically viable cash crop, puts farmers in a position where they must continue working in leopard-inhabited fields due to the absence of other sources of livelihood and lack of government support to grow alternative crops. While conservation is important, expecting these communities to bear its burdens without proper support and long-term solutions is problematic. Addressing human–leopard conflict therefore requires integrated, local-based approaches that simultaneously consider ecological, ethical, and socio-economic realities.

## Alternative solutions

IUCN has estimated a 24.5% decline in the Indian leopard population over the past three generations (IUCN Red List of Threatened Species 2023). So, while immuno-contraception may offer a non-lethal approach of managing local leopard populations, it risks reducing these numbers even further. Long-term coexistence will ultimately depend on addressing the ecological and socio-economic drivers of conflict rather than the population size alone. Simultaneously, communities in high-conflict zones often bear the highest cost of conservation through livelihood, livestock, and in some cases, life loss. Thus, long-term solutions require an integrated approach that balances coexistence and ecological sustainability with the socio-economic realities of a region.

Community-based initiatives such as ecotourism, conservation awareness programmes, and local stakeholder engagement have the potential to generate alternative livelihoods while fostering more positive attitude towards wildlife and strengthening coexistence. However, the benefits of such programmes are often unevenly distributed, depend on sustained institutional support, and may not be viable in every region. Similarly, strengthening compensation mechanisms through timely, transparent, and adequate payments can reduce resentment towards wildlife and improve tolerance among affected communities. Nevertheless, compensation alone is unlikely to resolve conflict if communities suffer recurring losses and payments are inadequate.

Measures that directly reduce opportunities for conflict must also be considered. For example, predator-proof livestock enclosures, night shelters, livestock insurance, and improved herding practices can substantially reduce livestock depredation, although their effectiveness depends on long-term maintenance and financial accessibility. Likewise, managing indirect drivers of conflict like abundance of stray dogs and feral pigs that offer easy prey through waste management may reduce the attractiveness of human landscapes to leopards. Again, implementing such solution would require coordinated action across multiple local municipalities and sustained community participation to keep the areas clean.

Long-distance translocation of surplus leopards may also be considered as a complementary measure, provided it is guided by ecological suitability and post-release monitoring. In many cases, translocation has produced mixed outcomes in large carnivores due to relocation stress, resulting in potential conflicts at release sites. Therefore, it should only be viewed as a complementary management strategy. Technological interventions can also complement broader coexistence strategies. AI-based camera traps can be used along with GPS collars used in national parks of India to track leopard movement and provide real-time alerts to local communities. But such technologies require substantial financial investment, human labour, long-term maintenance, and reliable communication infrastructure, which may limit their application in some conflict-prone regions.

Finally, addressing government and underlying drivers is also essential. In the Junnar landscape, forest fragmentation, expanding urbanisation, and widespread sugarcane cultivation have created suitable conditions for leopards. Promoting mixed farming, where farmers grow alternative crops and shorter sugarcane varieties, could reduce habitat suitability for leopards while diversifying farmers’ livelihoods. However, such transitions would require policy and financial incentives, including assured markets for alternative crops. Their implementation may also be challenging under current national policies promoting increased ethanol blending in petrol, which continues to promote sugarcane production to reduce fuel imports and carbon emissions (IBEF 2025). Similarly, better land-use planning through buffer zones, habitat restoration, and development regulations around forests can help reduce habitat fragmentation, although such measures often face political, administrative, and economic constraints. Finally, broader conservation priorities and resource allocation must be critically evaluated. Recent government investment in rewilding African cheetahs, a non-native species, in India demonstrates willingness to invest in conservation efforts. Comparable investments should be directed towards the conservation of native species, restoration of their habitats, mitigating human–leopard conflicts, and supporting communities living alongside wildlife, offering a more ecologically sound and socially inclusive approach to conservation.

## Data Availability

Data sharing is not applicable to this article as no new data were created or analysed in this study.
